# XGBoost-Based Feature Learning Method for Mining COVID-19 Novel Diagnostic Markers

**DOI:** 10.3389/fpubh.2022.926069

**Published:** 2022-06-22

**Authors:** Xianbin Song, Jiangang Zhu, Xiaoli Tan, Wenlong Yu, Qianqian Wang, Dongfeng Shen, Wenyu Chen

**Affiliations:** ^1^Department of Critical Care Medicine, Affiliated Hospital of Jiaxing University, Jiaxing, China; ^2^Department of Respiration, Affiliated Hospital of Jiaxing University, Jiaxing, China

**Keywords:** COVID-19, diagnostic markers, XGBoost, machine learning, principal component analysis

## Abstract

In December 2019, an outbreak of novel coronavirus pneumonia spread over Wuhan, Hubei Province, China, which then developed into a significant global health public event, giving rise to substantial economic losses. We downloaded throat swab expression profiling data of COVID-19 positive and negative patients from the Gene Expression Omnibus (GEO) database to mine novel diagnostic biomarkers. XGBoost was used to construct the model and select feature genes. Subsequently, we constructed COVID-19 classifiers such as MARS, KNN, SVM, MIL, and RF using machine learning methods. We selected the KNN classifier with the optimal MCC value from these classifiers using the IFS method to identify 24 feature genes. Finally, we used principal component analysis to classify the samples and found that the 24 feature genes could effectively be used to classify COVID-19-positive and negative patients. Additionally, we analyzed the possible biological functions and signaling pathways in which the 24 feature genes were involved by GO and KEGG enrichment analyses. The results demonstrated that these feature genes were primarily enriched in biological functions such as viral transcription and viral gene expression and pathways such as Coronavirus disease-COVID-19. In summary, the 24 feature genes we identified were highly effective in classifying COVID-19 positive and negative patients, which could serve as novel markers for COVID-19.

## Introduction

In December 2019, an epidemic of novel coronary pneumonia broke out in Wuhan, Hubei Province, China, which was considered by the World Health Organization to be a serious menace to the health of citizens of the world (1). This terrible communicable epidemic is caused by infection with the severe acute respiratory syndrome type 2 coronavirus (SARS-CoV-2), a sense single-stranded RNA virus (2). As a highly contagious virus, COVID-19 swept across the globe with alarming rapidity, leading to considerable losses to human society.

So far, the effective protection strategy against COVID-19 is to strengthen immunity ability and keep social distance (3). COVID-19 diagnosis is of great essence for the identification, isolation, and treatment of infectious objects (4). Existing detection methods include antibody assays that detect serum antiviral antibodies IgG and IgM, lateral chromatography assays that detect viral antigens, and real-time reverse transcriptase-polymerase chain reaction (qRT-PCR). The current gold standard for COVID-19 diagnosis is the application of qRT-PCR to verify the presence of SARS-CoV-2 RNA in the respiratory secretions of patients (5, 6). However, this detection method is not perfect because it is a complex test requiring a comprehensive and delicate infrastructure (5). And this method can only achieve accuracies of 30–60% in clinical application, which probably results in false-positive cases (7). More landmark diagnostic biomarkers are needed to detect COVID-19-positive patients with higher accuracy, reducing the false positive rate. Besides, exploring and developing new detection kits is of equal significance to facilitate the precise prevention and control of the epidemic.

Machine learning is applied extensively in biomedical applications, as well as COVID-19 diagnosis (8). Extreme Gradient Boosting (XGBoost) is a GBDT-based algorithm. Characterized by its high efficiency, flexibility, and portability, XGBoost is widely used in data mining, recommendation systems, and other fields (9). Zhang and GuoLiang (10) developed a machine learning algorithm for XPPA based on the XGBoost algorithm, which could be used to detect the effect of alterations in gene expression on aberrant p53 pathway activity. Athanasiou et al. (11) constructed a personalized risk prediction model for cardiovascular disease based on the XGBoost algorithm to predict the incidence of patients with cardiovascular disease. The follow-up results of 560 patients demonstrate that this predictive model has favorable performance (AUC = 71.13%), which is expected to provide new insights into clinical cardiovascular treatment. With a decoupling feature, XGBoost shows increased applicability, and it is a high-performance algorithm for modeling regarding the selection of loss functions on demand for classification and regression. Therefore, XGBoost is reliable to be applied in establishing a diagnostic, prognostic model based on patient features in clinical practice.

Here, we used the XGBoost algorithm to mine feature genes in the expression profiles of COVID-19 negative and positive samples, used a machine learning algorithm to construct MARS, KNN, SVM, MIL, and RF COVID-19 classifiers, and selected the best classifier using Iterated Function System (IFS) algorithm. Finally, the validity of this set of feature genes was verified by principal component analysis (PCA) and functional enrichment analysis, the results of which suggested the potential of the genes to be promising biomarkers for COVID-19.

## Materials and Methods

### Datasets Downloading and Processing

From the GEO database (https://www.ncbi.nlm.nih.gov/geo/), the dataset GSE152075 was downloaded, which contained gene expression data from throat swab samples from 430 COVID-19-positive patients and 54 negative patients. And the data acquisition platform was GPL18573 (Illumina NextSeq 500). Genes whose mean value of gene expression was below 1 and the maximum value of gene expression was below 5 were retained. The data were normalized using the “edgR” package (12).

### Model Training

To establish the link between behavioral features and classification, we implemented the XGBoost model using the machine learning algorithm XGBoost (https://xgboost.ai/). Key features were determined based on feature importance ranking and recursive elimination (9). XGBoost is a gradient advancing decision tree method whose objective function is defined as in Equation (1).


(1) $$\begin{array}{r} £\left(\phi \right)\;=\;\overset{n}{\underset{i\;=\;1}{\sum }}loss\left(y_{i{ŷ}_{i}}\right)\;+\;\overset{k}{\underset{k\;=\;1}{\sum }}\Omega \left(f_{k}\right)\;\left(1\right) \end{array}$$

In this formula, loss is the training loss, Ω (f) is the complexity of the tree, and k is the number of trees in the model. The model can be optimized by minimizing the objective function. For this reason, the additive model was used to calculate the training loss, and the Taylor expansion method was used to quickly optimize the prediction of the nth round of additive training. Greedy algorithm was used to determine the optimal complexity of the tree. In addition, we employed SMOTE for Bayesian optimization resampling of the training set due to unbalanced samples (13).

### Selecting the Optimal Classifier by IFS Method

After feature selection by XGBoost, IFS method was used to identify the genes of the optimal COVID-19 classifier. IFS incremental feature selection method (14) is an algorithm proposed by Liu and Setiono (15) to find the best or closest optimal feature subset. This algorithm is based on improved information gain, which can make the equivalent exchange of information. The algorithm selects a candidate feature set using an evaluation function unrelated to the classifier, applies the classifier to the candidate feature set, and selects a feature subset utilizing the accuracy of the classifier as a criterion.

A series of COVID-19 classifiers (16) was subsequently established using the python package “sklearn” in combination with algorithms such as MARS, KNN, SVM, MIL, and RF. The IFS curve was drawn based on 10-fold cross-validation, resulting in Matthews correlation coefficient (MCC) for each classifier, which is a parameter that can effectively reflect the classifier's effectiveness (17). The classifier with the most considerable MCC value is considered as the optimal classifier, and the genes involved in it are taken as the optimal feature genes.

### PCA and Sample Cluster Analysis

After the optimal COVID-19 classifier was determined, the PCA was performed on the data set using “FactoMineR” to extract the first and second principal components. PCA analysis is an unsupervised dimensionality reduction analysis method which can visually present the sample-to-sample method (18) by reducing the dimensionality of the dataset and reflecting the data to the representative dimensions PC_1 and PC_2. The effect of model classification was finally verified by pedigree cluster analysis of the samples using the “pheatmap” package (19).

### GO and KEGG Enrichment Analyses

GO biological function analysis and KEGG biological pathway analysis of feature genes were performed using “clusterProfiler”. GO and KEGG pathways with *p-*value < 0.05 were considered notably enriched (20).

## Results

### XGBoost and IFS Analysis Results

**Figure 1:**
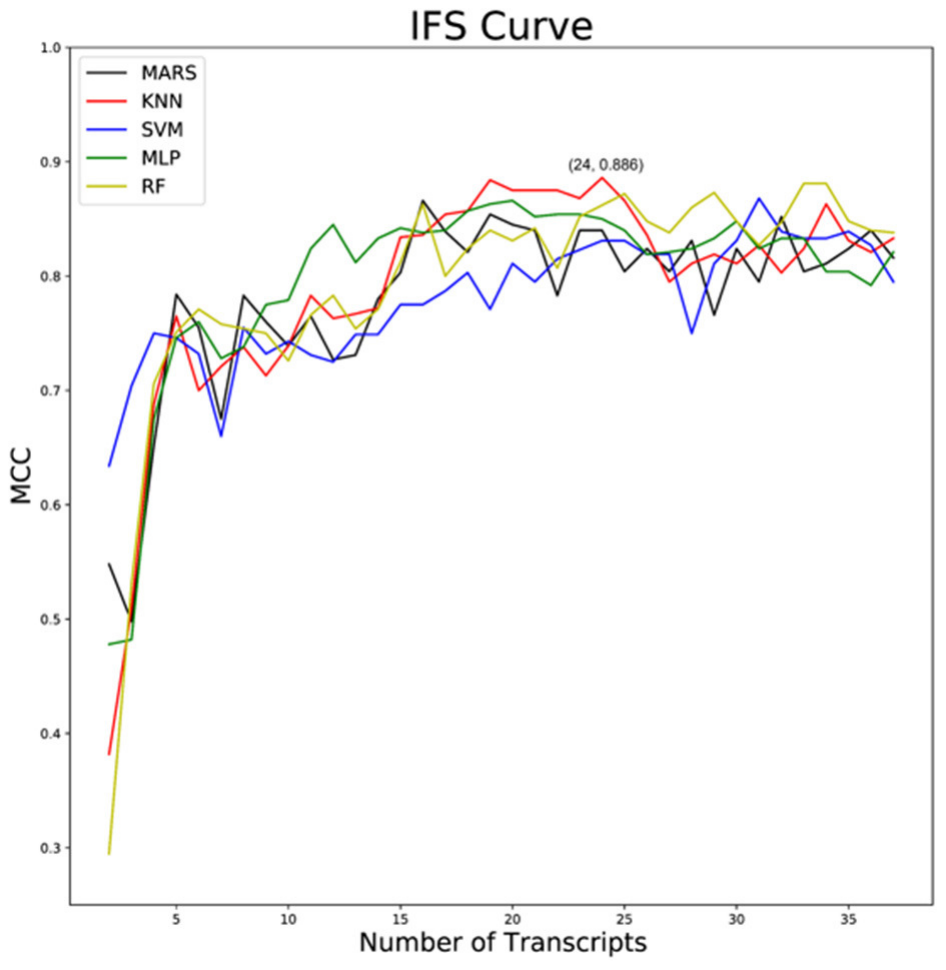
IFS curves of MARS, KNN, SVM, MLP, and RF classifiers. Black: MARS classifier; red: KNN classifier; blue: SVM classifier; green: MLP classifier; brownish-yellow: RF classifier; horizontal ordinate indicates the number of classifier genes and vertical ordinate represents MCC coefficient.

A total of 15,190 genes were obtained by the normalization of the gene expression data after the preprocessing of the dataset GSE152075. Thirty-seven feature genes that were ranked according to importance were obtained by XGBoost feature selection, which could distinguish sample types (Supplementary Table S1). And COVID-19 classifiers such as MARS, KNN, SVM, MLP, and RF were constructed based on these 37 feature genes. Then the best classifier was selected by IFS analysis, and we found that the KNN classifier was composed of 24 feature genes (IGFBP2, KRT8, RPLP0, XAF1, RPL13, OAS2, CES1, RPL4, EEF1G, NR2F6, RPS8, RPL10A, SNX14, C5orf15, TNFRSF19, CD24, ALAS1, CEP112, C9orf24, POLR2J3, AAMP, DUOX2, EMCN, RPL3) had the highest MCC value, MCC 0.886, sensitivity 0.986, specificity 0.907, and accuracy 0.977 (Figure 1).

### The Results of PCA Dimensionality Reduction Analysis and Sample Cluster Analysis

**Figure 2:**
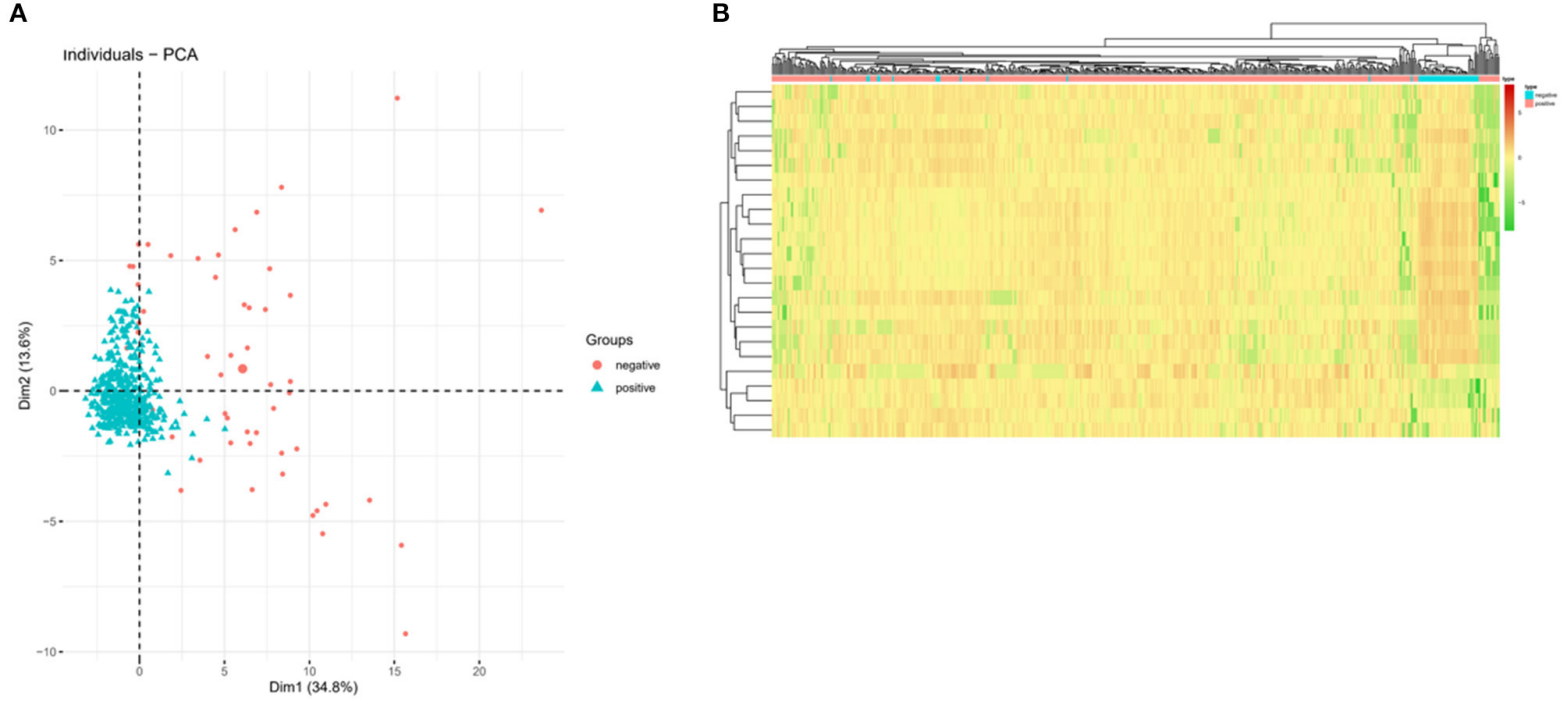
PCA and cluster heatmap analysis based on feature genes in the KNN classifier. **(A)** PCA shows the classification performance of the KNN classifier in COVID-19 negative (red) and positive (green) populations. **(B)** Cluster heatmap showing the expression of feature genes in the KNN classifier. Red indicates high expression and green indicates low expression.

PCA dimensionality reduction analysis was performed on the samples according to the expression of the 24-feature genes in the optimal KNN classifier, which showed that PCA analysis could classify COVID-19 in positive patients and negative persons (Figure 2A). In addition, we also plotted a cluster heatmap analyzing the expression of 24 feature genes in different populations. The results showed that the 24 feature genes in the KNN classifier could distinguish COVID-19 positive patients from normal healthy people (Figure 2B). These findings indicated that the 24 feature genes in the KNN classifier performed well in diagnosing COVID-19-positive patients and normal healthy people, showing superior diagnostic efficacy.

### The Results of GO and KEGG Enrichment Analyses

**Figure 3:**
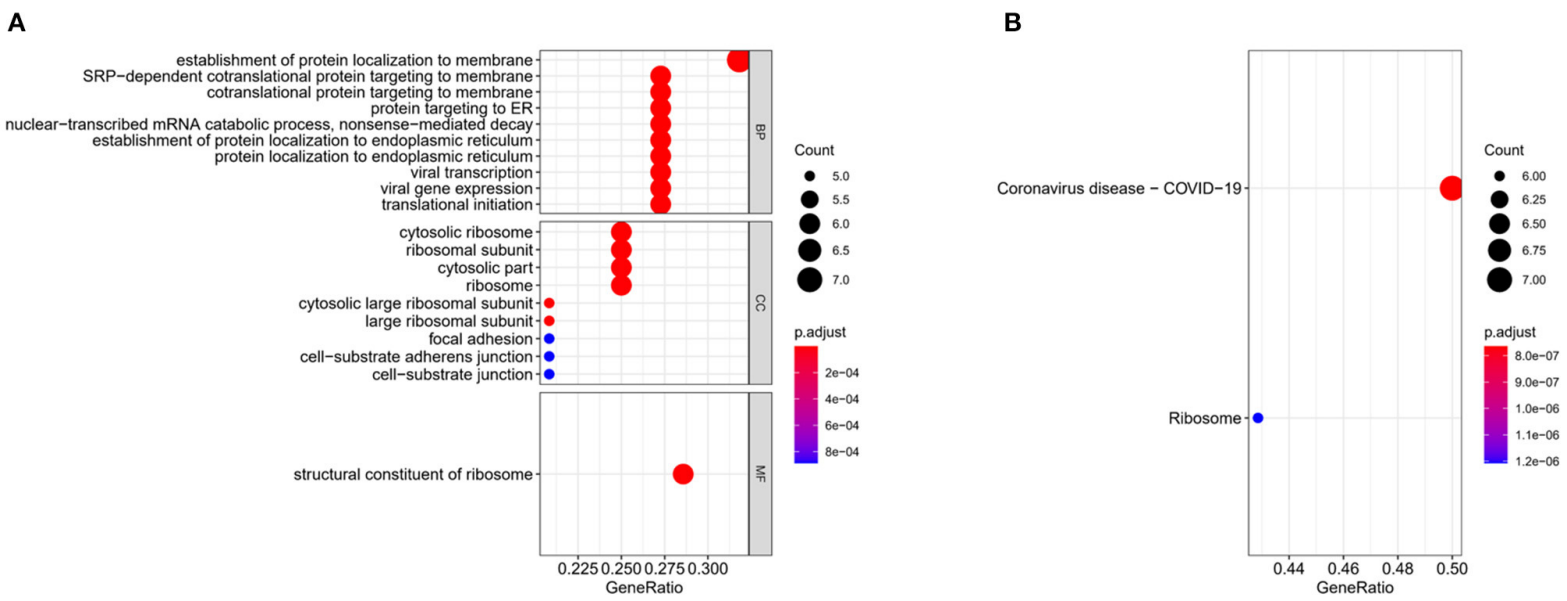
Gene enrichment analyses. **(A)** Bubble plots for GO enrichment analysis of 24 feature genes. **(B)** Bubble plots for KEGG enrichment analysis of 24 feature genes. The bubble size in the figure indicates the gene data in teams, and the color indicates the *p*-value, and the red the color, the smaller the *p*-value.

To identify the biological functions of feature genes and the signaling pathways involved, we performed enrichment analyses on the 24 feature genes. The GO analysis result showed that these genes were mainly enriched in biological functions such as viral transcription and viral gene expression (Figure 3A). KEGG biological pathway analysis showed gene enrichment on pathways such as Coronavirus disease-COVID-19 (Figure 3B). The selected feature genes were closely related to COVID-19 infection and its pathways.

## Discussion

Novel coronavirus pneumonia is a severe threat to global public health safety and brings enormous economic losses to human society. In this study, in order to identify new COVID-19 diagnostic biomarkers, we used the XGBoost algorithm to achieve feature selection and the IFS algorithm to determine the optimal classifier based on the throat swab expression profile data of COVID-19 positive and negative samples in the GEO database. After identifying the optimal feature genes, PCA, GO, and KEGG methods were used to verify whether the feature genes could be used as COVID-19 diagnostic biomarkers. First, we used the XGBoost algorithm to screen 37 feature genes from expression profiling data that could effectively distinguish COVID-19 positive from negative patients. Subsequently, KNN, SVM, MLP, and RF classifiers were constructed for the genes after feature selection, and the optimal classifier and its feature genes were selected based on the IFS method. Finally, we identified 24 feature genes, and based on the expression data of 24 feature genes, we performed PCA of the samples, and PCA results showed that PC_1 and PC_2 could effectively distinguish COVID-19 positive and negative samples. In addition, we performed GO and KEGG enrichment analyses of 24 feature genes, and the results showed that these feature genes were mainly gathered in biological functions such as viral transcription, viral gene expression, and pathways such as Coronavirusdisease-COVID-19. Therefore, combining all the results of bioinformatics analysis, the COVID-19 classifier of 24 feature genes was obtained in this study, while we reasonably speculated that the 24 feature genes screened in this study are expected to be novel diagnostic biomarkers for COVID-19.

Timely diagnosis of COVID-19 is essential for epidemic prevention and control, so identification of accurate diagnostic biomarkers is also an essential study for epidemic prevention and control. Feng et al. (21) constructed a machine learning diagnostic model using algorithms such as LASSO, AdaBoost, decision tree, and logistic regression based on patient clinical information to assist early COVID-19 diagnosis. The study by Kukar et al. (22) used machine learning methods to construct a COVID-19 diagnostic model based on blood routine parameters, which is complementary to chest CT and PT-PCR molecular diagnostics and improves COVID-19 diagnostic efficiency. Our study used the XGBoost algorithm to select feature genes in the expression profiles of throat swabs in positive patients, constructed classifiers such as MARS, KNN, SVM, MIL, and RF, and subsequently selected classifiers with optimal MCC values by the IFS method. At present, the conventional detection method of COVID-19 is nucleic acid detection, and the diagnostic biomarkers identified in this study are expected to improve the drawbacks of existing commercial nucleic acid detection kits and improve detection accuracy.

The optimal 24 feature genes, which were further analyzed by consulting the retrieved literature, we found that four genes (XAF1, OAS2, CES1, RPS8) have been reported in COVID-19. Gao et al. (23) found that XAF1 was abnormally strongly expressed in COVID-19 patients and positively correlated with the expression of ARS-CoV-2 invasion-related genes (ACE2, TMPRSS2, CTSB, and CTSL). In contrast, XAF1 was found to be associated with SARS infection by Park and Harris (24). A recent study found that OAS2 belongs to a subset of interferon-stimulated genes, and OAS2 can be regarded as a potential candidate for a drug target in COVID-19 therapy (25). The study by Li et al. (26) found that CES1 can hydrolyze tenofovir alafenamide (TAF), and effectively hydrolyzed TAF is significant for treating respiratory virus infection. In addition, Vastrad et al. (27) identified 10 SARS-CoV-2/COVID-19 diagnostic markers such as RPS8 using bioinformatics analysis methods. Also, several ribosomal proteins (RPL family members) contributing to protein synthesis were screened out. A report went that SARS-CoV-2 infection could result in ribosome dysfunction (28), giving us a hint that RPLs were affected at molecular degree. In combination with previous reports, it can be seen that some of the 24 feature genes are closely related to COVID-19. Finally, we performed GO and KEGG enrichment analyses, and the results showed that these feature genes were mainly enriched in biological functions such as viral transcription and viral gene expression as well as pathways such as Coronavirusdisease-COVID-19. We used bioinformatics methods to screen some genes that play an essential role in COVID-19 infection, which have also been reported as COVID-19-related genes in the existing literature. Even though it takes little time and hardly any money to detect COVID-19, some critical problems remain, like false positive case which concerns the public a lot. The combined various testing methods are urgently needed to remove false positive cases. Our study comes just in handy to provide some insights for developing novel strategy for COVID-19 diagnosis, which can definitely enrich current diagnostic tools.

## Conclusion

However, there are limitation in our study. First, this study is a retrospective study based on public databases, and no clinical samples are used to verify the performance of this classifier. Second, even if the mined genes were practically used for COVID-19 diagnosis, it is relatively costing to analyze 24 genes for one sample. Considering the limitations, we are planning to establish sample library and validate our model based on our collected samples. Overall, we mined optimal COVID-19 diagnostic biomarkers using machine learning algorithms, and our study, in combination with existing commercial nucleic acid detection kits, promises to improve COVID-19 detection accuracy.

## Data Availability Statement

The original contributions presented in the study are included in the article/Supplementary Material, further inquiries can be directed to the corresponding author.

## Author Contributions

XS and JZ: conceptualization. XT: methodology and investigation. WY: software and resources. XS, JZ, and WC: validation. WC and QW: formal analysis. DS: data curation and supervision. All authors: writing—original draft preparation and writing—review and editing. QW: visualization. XS: project administration. WC: funding acquisition. Funding acquisition: WC. All authors have read and agreed to the published version of the manuscript.

## Funding

This work was funded by Jiaxing Fight Novel Coronavirus Pneumonia Emergency Technology Attack Special Project in 2020 (No. 2020GZ30001), the Key Discipline of Jiaxing Respiratory Medicine Construction Project (No. 2019-zc-04, 2019-zc-12), General Scientific Research Project of Education Department of Zhejiang Province (No. Y202043729) and Jiaxing Key Laboratory of Precision Treatment for Lung Cancer.

## Conflict of Interest

The authors declare that the research was conducted in the absence of any commercial or financial relationships that could be construed as a potential conflict of interest.

## Publisher's Note

All claims expressed in this article are solely those of the authors and do not necessarily represent those of their affiliated organizations, or those of the publisher, the editors and the reviewers. Any product that may be evaluated in this article, or claim that may be made by its manufacturer, is not guaranteed or endorsed by the publisher.

## Supplementary Material

Supplementary Table S1 Thirty-seven feature genes obtained based on the XGBoost algorithm.

## References

[1] Zhu N Zhang D Wang W Li X Yang B Song J. A novel coronavirus from patients with pneumonia in China 2019. N Engl J Med. (2020) 382:727–33. 10.1056/NEJMoa2001017 31978945 PMC7092803

[2] Yuce M Filiztekin E Ozkaya KG. COVID-19 diagnosis-a review of current methods. Biosens Bioelectron. (2021) 172:112752. 10.1016/j.bios.2020.112752 33126180 PMC7584564

[3] Koo JR Cook AR Park M Sun Y Sun H Lim JT. Interventions to mitigate early spread of SARS-CoV-2 in Singapore: a modelling study. Lancet Infect Dis. (2020) 20:678–88. 10.1016/S1473-3099(20)30162-6 32213332 PMC7158571

[4] Salathe M Althaus CL Neher R Stringhini S Hodcroft E Fellay J. COVID-19 epidemic in Switzerland: on the importance of testing, contact tracing and isolation. Swiss Med Wkly. (2020) 150:w20225. 10.4414/smw.2020.20225 32191813

[5] Loeffelholz MJ Tang YW. Laboratory diagnosis of emerging human coronavirus infections - the state of the art. Emerg Microbes Infect. (2020) 9:747–56. 10.1080/22221751.2020.1745095 32196430 PMC7172701

[6] Corman VM Landt O Kaiser M Molenkamp R Meijer A Chu DK. Detection of 2019 novel coronavirus (2019-nCoV) by real-time RT-PCR. Euro Surveill. (2020) 25:45. 10.2807/1560-7917.ES.2020.25.3.2000045 31992387 PMC6988269

[7] Ai T Yang Z Hou H Zhan C Chen C Lv W. Correlation of chest CT and RT-PCR testing for coronavirus disease 2019 (COVID-19) in China: a report of 1014 CASES. Radiology. (2020) 296:E32–40. 10.1148/radiol.2020200642 32101510 PMC7233399

[8] Usman M Gunjan VK Wajid M Zubair M Siddiquee KN. Speech as A Biomarker for COVID-19 detection using machine learning. Comput Intell Neurosci. (2022) 2022:6093613. 10.1155/2022/6093613 35444694 PMC9014833

[9] Chen T Guestrin C. XGBoost: a scalable tree boosting system. In: Proceedings of the 22nd ACM SIGKDD International Conference on Knowledge Discovery and Data Mining. San Francisco, CA: Association for Computing Machinery (2016). p. 785–94.

[10] Zhang A Liu CR Lin GL. P53 pathway activate detection based on machine learning: The modified XGBoost-based method of pan-cancer pathway activity detection in the cancer genome atlas. In: CCEAI 2021: 5th International Conference on Control Engineering and Artificial Intelligence. New York, NY: Association for Computing Machinery (2021).

[11] Athanasiou M Sfrintzeri K Zarkogianni K Thanopoulou AC Nikita KS. An explainable XGBoost-based approach towards assessing the risk of cardiovascular disease in patients with Type 2 Diabetes Mellitus 2020. In: IEEE 20th International Conference on Bioinformatics and Bioengineering. Cincinnati, OH: IEEE (2020).

[12] Robinson MD McCarthy DJ Smyth GK. edgeR: a Bioconductor package for differential expression analysis of digital gene expression data. Bioinformatics. (2010) 26:139–40. 10.1093/bioinformatics/btp616 19910308 PMC2796818

[13] Nakamura M Kajiwara Y Otsuka A Kimura H. LVQ-SMOTE - learning vector quantization based synthetic minority over-sampling technique for biomedical data. BioData Min. (2013) 6:16. 10.1186/1756-0381-6-16 24088532 PMC4016036

[14] Gui T Dong X Li R Li Y Wang Z. Identification of hepatocellular carcinoma-related genes with a machine learning and network analysis. J Comput Biol. (2015) 22:63–71. 10.1089/cmb.2014.0122 25247452

[15] Liu H Setiono R. Incremental feature selection. Appl Intellig. (1998) 9:217–30. 10.1023/A:1008363719778

[16] Yang F Wang X Ma H Li J. Transformers-sklearn: a toolkit for medical language understanding with transformer-based models. BMC Med Inform Decis Mak. (2021) 21:90. 10.1186/s12911-021-01459-0 34330244 PMC8323195

[17] Chicco D Jurman G. The advantages of the Matthews correlation coefficient (MCC) over F1 score and accuracy in binary classification evaluation. BMC Genomics. (2020) 21:6. 10.1186/s12864-019-6413-7 31898477 PMC6941312

[18] Yang L Qin Y Jian C. Screening for core genes related to pathogenesis of Alzheimer's disease. Front Cell Dev Biol. (2021) 9:668738. 10.3389/fcell.2021.668738 33968940 PMC8101499

[19] Tal Galili AOC. Jonathan Sidi, Carson Sievert. heatmaply: an R package for creating interactive cluster heatmaps for online publishing. Bioinformatics. (2017) 34:1600–2. 10.1093/bioinformatics/btx657 29069305 PMC5925766

[20] Yu G Wang LG Han Y He QY. clusterProfiler: an R package for comparing biological themes among gene clusters. OMICS. (2012) 16:284–7. 10.1089/omi.2011.0118 22455463 PMC3339379

[21] Feng C Wang L Chen X Zhai Y Zhu F Chen H. A novel artificial intelligence-assisted triage tool to aid in the diagnosis of suspected COVID-19 pneumonia cases in fever clinics. Ann Transl Med. (2021) 9:201. 10.21037/atm-20-3073 33708828 PMC7940949

[22] Kukar M Guncar G Vovko T Podnar S Cernelc P Brvar M. COVID-19 diagnosis by routine blood tests using machine learning. Sci Rep. (2021) 11:10738. 10.1038/s41598-021-90265-9 34031483 PMC8144373

[23] Gao X Liu Y Zou S Liu P Zhao J Yang C. Genome-wide screening of SARS-CoV-2 infection-related genes based on the blood leukocytes sequencing data set of patients with COVID-19. J Med Virol. (2021) 93:5544–54. 10.1002/jmv.27093 34009691 PMC8242610

[24] Park A Harris LK. Gene expression meta-analysis reveals interferon-induced genes associated with SARS infection in lungs. Front Immunol. (2021) 12:694355. 10.3389/fimmu.2021.694355 34367154 PMC8342995

[25] Prasad K Khatoon F Rashid S Ali N AlAsmari AF Ahmed MZ. Targeting hub genes and pathways of innate immune response in COVID-19: a network biology perspective. Int J Biol Macromol. (2020) 163:1–8. 10.1016/j.ijbiomac.2020.06.228 32599245 PMC7319641

[26] Li J Liu S Shi J Zhu HJ. Activation of tenofovir alafenamide and sofosbuvir in the human lung and its implications in the development of nucleoside/nucleotide prodrugs for treating SARS-CoV-2 pulmonary infection. Pharmaceutics. (2021) 13:656. 10.3390/pharmaceutics13101656 34683949 PMC8540046

[27] Vastrad B Vastrad C Tengli A. Bioinformatics analyses of significant genes, related pathways, and candidate diagnostic biomarkers and molecular targets in SARS-CoV-2/COVID-19. Gene Rep. (2020) 21:100956. 10.1016/j.genrep.2020.100956 33553808 PMC7854084

[28] Lapointe CP Grosely R Johnson AG Wang J Fernandez IS Puglisi JD. Dynamic competition between SARS-CoV-2 NSP1 and mRNA on the human ribosome inhibits translation initiation. Proc Natl Acad Sci U S A. (2021) 118:118. 10.1073/pnas.2017715118 33479166 PMC8017934

