# Supplementary figures and images for: XGBoost-Based Feature Learning Method for Mining COVID-19 Novel Diagnostic Markers
Source: Front Public Health. 2022 Jun 22;10:926069. doi: 10.3389/fpubh.2022.926069 (PMC9256927; doi:10.3389/fpubh.2022.926069)

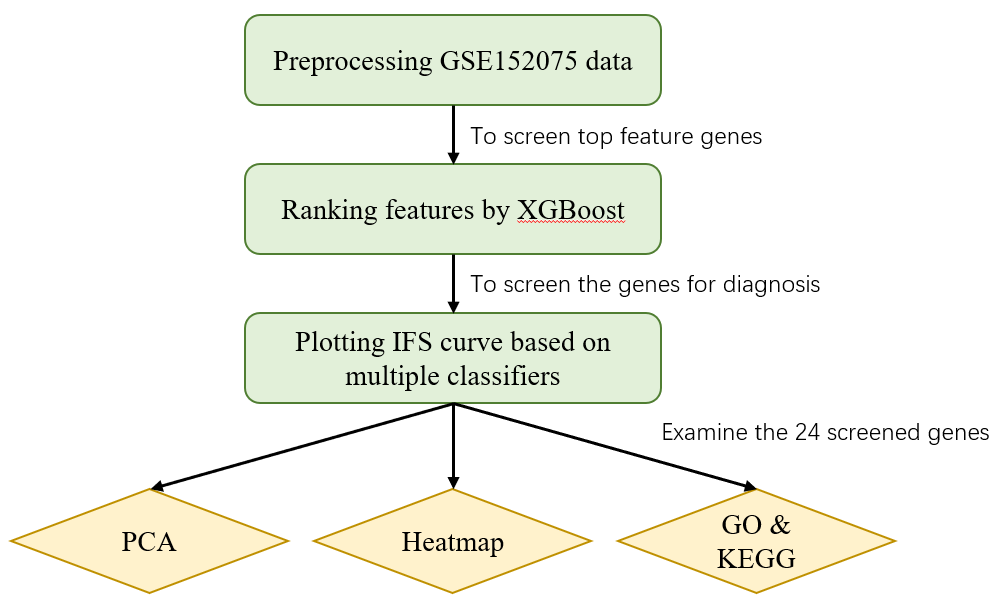

Supplement: Supplementary file 1 [file Image_1.TIF]
